# Noise after total knee arthroplasty has limited effect on joint awareness and patient-reported clinical outcomes: retrospective study

**DOI:** 10.1186/s12891-020-3134-7

**Published:** 2020-02-21

**Authors:** Hiroto Taniguchi, Masafumi Itoh, Nobuyuki Yoshimoto, Junya Itou, Umito Kuwashima, Ken Okazaki

**Affiliations:** 1 Department of Orthopaedic Surgery, Yachiyo Medical Center, Tokyo Women’s Medical University, Chiba, Japan; 2 Department of Orthopaedic Surgery, Tokyo Women’s Medical University, 8-1 Kawadacho, Shinjuku, Tokyo, Japan

**Keywords:** Total knee arthroplasty, Patient-reported outcome measures, Joint awareness, Patient satisfaction, Noises

## Abstract

**Background:**

Some patients complain of noise after total knee arthroplasty (TKA). Controversy still exists on how the noise affects the clinical outcomes, including joint awareness, after TKA. The Forgotten Joint Score—12 (FJS-12) measures the clinical outcomes focusing on joint awareness after surgery. The Knee Society Scoring System—2011 (KSS-2011) includes questionnaires for satisfaction, expectation, and functional activities. The aim of this study is to clarify the relationship among FJS-12, KSS-2011, and the noise. Furthermore, the relationship between FJS-12 and KSS-2011 was validated.

**Methods:**

Using FJS-12 and KSS-2011, 295 knees from 225 patients who underwent TKA were retrospectively evaluated. Noise perception was evaluated by a questionnaire with five grades, a method that follows the questionnaire form of FJS-12 (“Are you aware of the noise of your artificial joint?”; never, almost never, seldom, sometimes, mostly). Correlations among FJS-12, KSS-2011, and noise were analyzed. The patients were divided into four groups based on the mechanism of their implant [cruciate retaining, posterior stabilized, cruciate sacrificed, and bicruciate stabilized (BCS)]. FJS-12, KSS-2011, and noise were compared among the groups.

**Results:**

A strong correlation was found between FJS-12 and total score of KSS-2011 (0.70; *P* < 0.001). FJS-12 correlated with KSS-2011 subcategories of “symptoms,” “satisfaction,” and “standard activities,” with correlation coefficients at approximately 0.60. Noise had weak correlations with FJS-12 (0.28; *P* < 0.001) and KSS-2011 (0.20 *P* < 0.001). In comparing the TKA mechanisms, BCS had remarkably better KSS-2011 and greater movement range but worse noise scores.

**Conclusions:**

Noise perception after TKA had limited effect on joint awareness and clinical outcomes. FJS-12 correlated strongly with KSS-2011 and associated with satisfaction, residual symptoms, and daily activities, as assessed by KSS-2011 subscores.

**Trial registration:**

This study was approved by the Medical Ethical Committee of the Tokyo Women’s Medical University (approval number: 4681 on March 2, 2018).

## Background

Total knee arthroplasty (TKA) is a standard surgical treatment for advanced-stage knee osteoarthritis [1]. Regarding the method used to evaluate its outcome, physician-derived scores had an important role in the past. Nevertheless, inconsistency was found between physician- and patient-derived scores [2]. In addition, several reports revealed that patient satisfaction after TKA is not high [3–5]. Therefore, patient-reported outcome measures (PROMs), such as the Western Ontario and McMaster Universities Osteoarthritis Index (WOMAC) [6], Knee Injury and Osteoarthritis Outcome Score (KOOS) [7], and Oxford Knee Score (OKS) [8], are used frequently to evaluate postoperative outcomes after TKA.

The Knee Society also revised the evaluation method for TKA to PROMs in 2011, and it was launched as the Knee Society Scoring System 2011 (KSS-2011) [9, 10]. This scoring system includes questionnaires related to the category of satisfaction, which directly evaluates patient satisfaction after TKA. Furthermore, questionnaires about the knee condition when more active patients enjoy leisure activities and sports were included to daily activities.

The Forgotten Joint Score-12 (FJS-12) is a PROM developed in 2012 [11]. Good joints are considered “no awareness,” and the FJS-12 is useful to evaluate joint awareness. FJS-12 correlated with PROMs associated with knee joints, such as WOMAC, KOOS, and OKS [11–15], and it is effective for outcome evaluation after TKA. The FJS-12 has an advantage in that it has 12 questions and fewer ceiling and floor effects. Nevertheless, the relationship between FJS-12 and KSS-2011, which are the most recently developed PROMs used to evaluate postoperative TKA performance, has not been validated. In particular, because KSS-2011 is the only PROM that contains a “patient satisfaction” category, the relationship between FJS-12 and subcategories of KSS-2011, including “patient satisfaction,” should be evaluated.

In addition, joint noise frequently occurs after TKA. Nam et al. [16] reported that noise after TKA is related to residual symptoms, whereas Kuriyama et al. [17] reported that noise was not correlated with patient satisfaction after TKA. Noise may be related to joint awareness after TKA. Nevertheless, no enough information is available on whether the noise after TKA is related to patient-reported outcomes, including joint awareness.

The aim of this study is to reveal how joint awareness correlates with knee symptoms, functions, postoperative satisfaction, and noise perception after TKA. To assess these questions, the relationship between FJS-12 and KSS-2011 and the perception of noise were evaluated. Furthermore, the relationship between PROMs (FJS-12, KSS-2011, and noise perception) and factors, such as TKA mechanism and range of movement (ROM), was assessed.

## Methods

A total of 476 patients (598 knees) who underwent primary TKA from January 2007 to November 2017 at our hospital and related institutions and for whom ≥1 year had elapsed after TKA were enrolled in this study. After excluding patients who died or whose implants were removed owing to loosening or infection, the questionnaire survey, including FJS-12 and KSS-2011, was mailed to 451 patients (566 knees). We used the validated Japanese versions of the questionnaires and signed license agreements with the copyright owners.In addition, the noise in the knee after TKA was assessed via a questionnaire asking if the patients feel any noise during the activity of daily life. Noise scoring was defined on the basis of a 5-point scale: never feel (4 points), feel almost never (3 points), feel seldom (2 points), feel sometimes (1 point), and feel mostly (0 points). This questionnaire used the same expression as that of FJS-12. In patients who underwent bilateral surgery, the responses for two knees on each side were obtained. Valid responses for FJS-12, KSS, and noises were obtained from 289 patients (372 knees, questionnaire collection rate, 65.7%).From the medical records, age at operation, body mass index (BMI) at the time of the survey, and preoperative and postoperative ROM were examined. Hence, in 225 (295 knees) patients, valid data on all items of FJS-12, KSS2011, noise, preoperative ROM, and postoperative ROM were obtained.This study was approved by the institutional review board of our institution (approval number: 4681).

The 11 models of TKA implant used in this research were classified based on the mechanism as posterior stabilized (PS; 91), cruciate retaining (CR; 78), cruciate sacrificed (without post-cam mechanism) (CS; 102), and bi-cruciate stabilized (BCS; 24). The models used were Legion® PS (39 knees; Smith & Nephew, Memphis, TN, USA), Legion® CR (11 knees; Smith & Nephew), NexGen® LPS (31 knees; Zimmer, Warsaw, IN, USA), NexGen® CR (12 knees; Zimmer), Genesis II® PS (19 knees; Smith & Nephew), Genesis II® CR (31 knees; Smith & Nephew), Persona® CR (12 knees; Zimmer), Advance® CR (14 knees; Wright, Memphis, TN, USA), LCS® rotation platform CS (81 knees; Depuy), GMK Sphere® CS (19 knees; Medacta, Strada Regina, Switzerland), and Journey II® BCS (24 knees; Smith & Nephew). The TKA models were chosen by the surgeons’ preferences at operation.

### Statistical analysis

For statistical examination, Cronbach’s *α* was used to evaluate the internal consistency of FJS-12, and > 0.9 indicated that the scale is reliable. The Shapiro–Wilk test was used to evaluate normal distribution. The ceiling or floor effects were considered to be present if more than 15% of the respondents achieved the highest or lowest possible score [13, 18]. Because the Shapiro–Wilk test revealed that FJS-12 and KSS-2011 did not have normal distribution, the correlation between FJS-12 and KSS-2011 was examined using Spearman’s correlation coefficient. Noise was examined also for correlation with FJS-12 or KSS-2011. In addition, FJS-12, KSS-2011, and noise examined the correlation between the patient characteristics (age at operation and BMI at questionnaire collection) and ROM pre- and postoperatively. Regarding the TKA mechanism, differences among the mechanisms in patient characteristics, ROM, FJS-12, KSS-2011, and noise were examined using analysis of variance (ANOVA). Regarding ANOVA, power analysis was performed and detection power was calculated. Multiple comparisons were performed with the Steel–Dwass test. JMP Pro 14.0.0 was used in statistical processing, and the rejection area was set to 5%.

## Results

There were 180 female and 45 male subjects. The right side was 163 and the left side was 132. The demographic data for the subject’s characteristics are found in Table 1.

**Table 1:** Characteristics for validation sample

Factors	Mean (S.D.)	Range
Age at surgery time	72.6 (7.6)	51–89
BMI	26.3 (4.7)	16.6–42.1
Preoperative ROM	109.3 (20.3)	40–145
Postoperative ROM	121.9 (13.1)	75–150
Time since surgery (month)	49.5 (30.0)	12–139

*S.D.* standard deviation, *BMI* body mass index, *ROM* range of motion

**Fig. 1:**
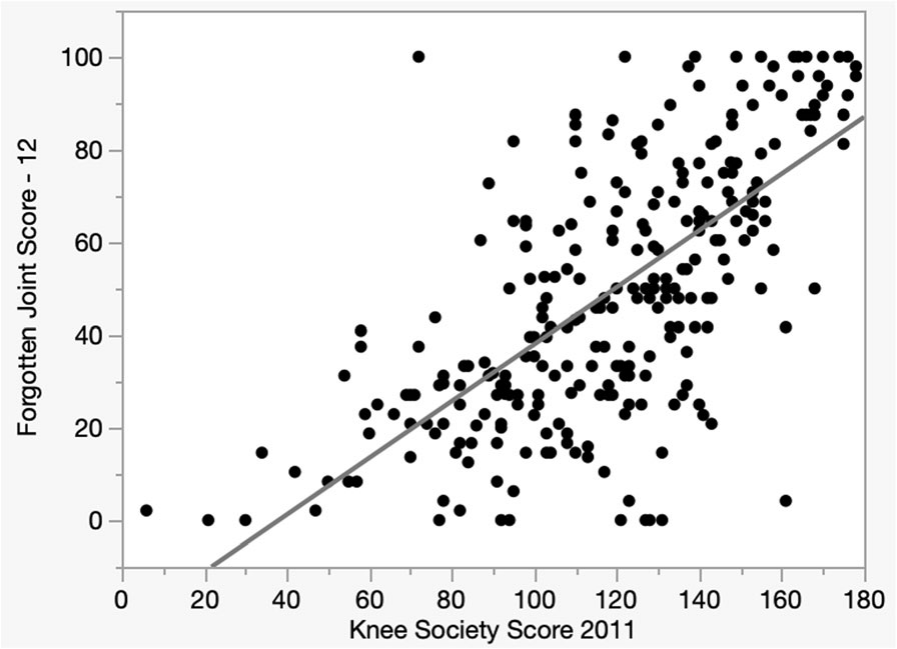
The graph reveals the relationship between the Forgotten Joint Score—12 (FJS-12) and the 2011 Knee Society Score (KSS-2011). Linear regression analysis revealed a strong correlation between FJS-12 and KSS-2011 scores with a correlation coefficient at 0.70 (95% confidence interval, 0.65–0.75; *P* < 0.001)

FJS-12 had an average score of 49.8 [standard deviation (SD), 28.4]. The ceiling effect was 15.6%, and the floor effect was 11.9% (Table 2). Regarding the average value of each of the 12 items, question 8 (“when you are standing up from a low-sitting position?”) had the worst score (average, 3.67 points). Regarding response rate, several patients (14.5%) did not answer question 12 (“when you are doing your favorite sport?”; Table 3). The Cronbach’s *α* value was 0.947. KSS-2011 had an average of 119.3 (SD, 32.4). The ceiling effect, floor effect, and the results of each item are found in Tables 2 and 4. A strong correlation was found between FJS-12 and the total scores of KSS-2011 (0.70; 0.65–0.75; *p* < 0.001 in 95% confidence interval; Fig. 1). The correlation between FJS-12 and the subscores of KSS-2011 by category is found in Table 4. A positive correlation was observed in all items, and correlation coefficients of approximately 0.60 were observed for “symptoms,” “patient satisfaction,” “functional activities,” and “standard activities.” Noise had weak correlations with FJS-12 (0.28; *p* < 0.001) and KSS-2011 (0.20; *p* < 0.001). Age, BMI, and ROM had also no effect on FJS-12, KSS-2011, and noise (Table 5).

**Table 2:** Postoperative Results of FJS-12, KSS-2011, and Noise

	Mean (S.D.)	Range	Ceiling effect	Floor effect
FJS-12	49.8 (28.4)	0–100	15.59 % (46/295)	11.86 % (35/295)
KSS-2011	119.3 (32.4)	6–178	16.61 % (49/295)	0.67 % (2/295)
Noise	3.1 (1.3)	0–4	56.61 % (167/295)	1.69 % (5/295)

**Table 3:** Results of Forgotten Joint Score—12

	Questionnaires	Mean (S.D.)	Missing rate	
Are you aware of your artificial joint				
1	in bed at night?	2.30 (1.42)	0.00%	(0/295)
2	when you are sitting on a chair for more than 1 h?	2.40 (1.41)	0.00%	(0/295)
3	when you are walking for more than 15 min?	2.88 (1.51)	0.00%	(0/295)
4	when you are taking a bath/shower?	2.51 (1.47)	0.00%	(0/295)
5	when you are traveling in a car?	2.33 (1.34)	0.34%	(1/295)
6	when you are climbing stairs?	3.45 (1.44)	0.00%	(0/295)
7	when you are walking on uneven ground?	3.40 (1.41)	1.02%	(3/295)
8	when you are standing up from a low-sitting position?	3.67 (1.35)	0.00%	(0/295)
9	when you are standing for long periods of time?	3.29 (1.48)	0.34%	(1/295)
10	when you are doing housework or gardening?	3.15 (1.42)	0.00%	(0/295)
11	when you are taking a walk/hiking?	3.30 (1.46)	2.71%	(8/295)
12	when you are doing your favorite sport?	3.47 (1.48)	14.58%	(43/295)
*Score*	*never 1, almost never 2, seldom 3, sometimes 4, mostly 5*			

*S.D.* standard deviation

**Table 4:** Postoperative KSS-2011 Subscores and Correlations to FJS-12

KSS-2011 Subscores	Mean (S.D.)	Correlation coefficient	*p* value
I. Symptoms score	19.5 (5.7)	0.61	< 0.001
II. Satisfaction score	27.7 (8.3)	0.63	< 0.001
III. Expectation score	10.6 (3.1)	0.44	< 0.001
IV. Functional activities score	61.5 (21.3)	0.60	< 0.001
i. Walking and standings	19.0 (9.3)	0.41	< 0.001
ii. Standard activities	23.1 (5.6)	0.65	< 0.001
iii. Advanced activities	11.3 (6.6)	0.48	< 0.001
iv. Discretionary activities	8.1 (5,3)	0.45	< 0.001

**Table 5:** Correlation Coefficients Among FJS-12, KSS-2011, and Noise

	Correlation to FJS-12		Correlation to KSS2011		Correlation to Noise	
	SC	*p* value	SC	*p* value	SC	*p* value
Age	−0.06	*p = 0.266*	−0.20	*p < 0.001*	0.14	*p = 0.017*
BMI	0.04	*p = 0.519*	−0.05	*p = 0.406*	−0.09	*p = 0.126*
Preoperative ROM	−0.02	*p = 0.712*	0.01	*p = 0.895*	−0.12	*p = 0.042*
Postoperative ROM	0.03	*p = 0.625*	0.14	*p = 0.016*	− 0.15	*p = 0.008*
Noise	0.28	*p < 0.001*	0.20	*p < 0.001*		

*FJS*-12 forgotten joint score; 12, *KSS*-2011 2011 knee society score, *SC* Spearman’s correlation coefficient, *BMI* body mass index, *ROM* range of motion

Moreover, FJS-12, KSS-2011, postoperative ROM, and noise were examined based on the mechanisms (CS, CR, PS, and BCS), and no remarkable differences were observed in FJS-12. Nevertheless, a remarkable difference was found in KSS-2011, postoperative ROM, and noise. Among the four mechanisms in KSS-2011 and postoperative ROM, BCS had the best performance, whereas noise had the lowest score. Multiple comparisons revealed that BCS had remarkably better KSS-2011 scores than PS, greater ROM than most of the other mechanisms, but lower noise score than CR (Table 6). Power analysis revealed more than 85% statistical power in detecting the difference among the groups with this sample number in each category of examination.

**Table 6:** Results of ANOVA by Mechanism of TKA

	Mean (S.D.)				*p* value	Statistical power	Significant difference pair
	BCS	CR	CS	PS			
	*n* = 24	*n* = 78	*n* = 102	*n* = 91			
Age	71.8 (4.9)	72.6 (7.2)	74.3 (8.9)	70.9 (4.2)	0.016		PS–CS
BMI	26.0 (4.2)	25.6 (4.4)	26.0 (4.4)	27.0 (5.5)	0.373		
Preoperative ROM	117.7 (15.3)	111.7 (17.5)	104.9 (22.5)	109.9 (20.2)	0.016	0.92	
Postoperative ROM	129.0 (9.6)	122.3 (11.1)	117.4 (14.5)	124.6 (12.4)	< 0.001	0.99	PS–CS CS– BCS
FJS-12	57.2 (27.1)	55.6 (24.5)	51.2 (31.4)	41.3 (26.5)	0.004	0.89	PS–CR
KSS 2011	140.3 (24.8)	124.9 (26.5)	120.1 (33.4)	108.0 (33.9)	< 0.001	0.99	PS–BCS PS–CR CS–BCS
Noise	2.7 (1.5)	3.3 (1.1)	3.2 (1.2)	2.9 (1.3)	0.027	0.86	

## Discussion

This study revealed that FJS-12 and KSS-2011 had a strong correlation in patients who underwent TKA, confirming the validation of the two most recent scoring system with each other. Although noise was hypothesized to have an effect on joint awareness, only a weak correlation was found with FJS-12 and KSS-2011. In fact, BCS had the worst score in noise, but the best score in KSS-2011, and the greatest ROM. Noise is suggested to have a limited effect on joint awareness and clinical outcomes. Residual symptoms and joint functions rather than noise perception might be important for joint awareness after TKA.

Of the KSS-2011 subscores, correlation coefficients of approximately 0.60 were found for “symptoms,” “patient satisfaction,” and “functional activities” for FJS-12. It became clear that joint awareness was correlated with postoperative pain and activities of daily living. Although FJS-12 does not directly question patient satisfaction, it also can evaluate patient satisfaction because it also is associated with patient satisfaction from KSS-2011, which directly asks for satisfaction. In addition, as the functional activities of KSS-2011 are divided into subcategories (“walking and standing,” “standard activities,” “advanced activities,” and “discretional activities”), our study indicated a significant correlation between FJS-12 and standard activities. Therefore, joint awareness was influenced by the difficulty level of routine activities, such as walking on an uneven surface, turning or pivoting, climbing up or down a stairs, rising from a low chair, and stepping to the side. FJS-12 is a simple questionnaire with only 12 items and has few ceiling and floor effects. Therefore, FJS-12 is a useful tool to evaluate residual symptoms, daily activity performance, and patient satisfaction after TKA.

Noise was expected to be correlated with joint awareness. Nevertheless, it had only a weak correlation with FJS-12 and KSS-2011. Although the patients with TKA are aware of a noise, some studies have revealed that it has minimal influence on patient satisfaction if the functional aspects, such as ROM, are good [17]. The previous study was done with a TKA model using a unique tricondylar mechanism; therefore, the noise perception influence on PROMs for patients with conventional TKA models needed to be elucidated. In this study of TKA mechanisms, a remarkable difference was found between KSS-2011 and postoperative ROM, and the BCS results were good. A remarkable difference was confirmed regarding noise, and the BCS score was the lowest. Nam et al. [16] reported that noise was recognized in 27% of the patients and the likelihood of noise generation was different among the TKA mechanisms; PS design was the greatest, followed by rotating-platform, sex-specific, and CR factors. Furthermore, patient-recognized noise was reportedly associated with residual symptoms, such as difficulty in getting in and out of cars, limp, stiffness, or swelling. The inconsistency of the findings between the previous studies and our study can be caused by the difference in assessing noise frequency. The previous studies categorized subjects into two groups based on with or without noise, whereas our study used an ordinal scale. From our findings, the noise score had remarkable correlations both with FJS-12 and KSS-2011, but the correlation coefficients were small. It was suggested that even if noise was recognized, patient satisfaction postoperatively would be high if functions, such as the ROM, were good. Therefore, noise itself had a limited effect on joint function, overall satisfaction, and joint awareness after TKA. Nevertheless, it should be encouraged to find better implant design and surgical techniques to reduce the noise with high function after TKA.

This study had several limitations. First, there was a question with low response rate in FJS-12. As reported in previous studies [19, 20], the response rate for item 12 was particularly low. It may be reasonable to consider that some patients who underwent TKA were relatively inactive. Nevertheless, FJS-12 has been confirmed to be valid with a few missing items [11]. In addition, the collection rate of scores was low (65.7%). The reasons for this are as follows: in the collection process of the questionnaires by mail, we found some patients with newly confirmed death, unresponsiveness owing to dementia, and serious disabilities other than knee disabilities. In addition, some patients relocated and returned invalid responses. The process of eliminating the invalid responses would improve the response reliability even though it would reduce the response rate. Furthermore, the effect of bias owing to the low responsive rate on the results is estimated to be small because it is a cross-sectional study that examines the correlation within one sample and the final number of respondents was over 200.Second, this was a retrospective cross-sectional study, and there were time variations in data collection in the answers of FJS-12. In fact, some studies have found that the FJS-12 scores change over time [21]. The differences in questionnaire sampling time might have influenced the results. Nevertheless, we recruited patients for whom at least 1 year had passed postoperatively. Third, various TKA models had been used. Although the mechanisms can be categorized into four systems, several models remained within the same category. Furthermore, preoperative patient conditions were not matched among the groups. Therefore, this study did not conclude that BCS was superior in clinical outcomes than in any other TKA mechanisms. We did not attempt to investigate which mechanism had the best performance. The point of this analysis was to show that some TKA models exhibit greater noise generation while showing higher KSS-2011 score. Thus, this finding suggested that noise perception has a limited effect on clinical outcomes. Lastly, the noise score used in this study has not been validated previously. Because there is no score as a reference to evaluate the noise perception, the same questionnaire expression method as the FJS-12 was used to evaluate noise.

## Conclusions

A strong correlation was found between FJS-12 and KSS-2011. In addition, FJS-12 was associated with the subcategories of KSS-2011 for symptoms, patient satisfaction, and standard activities. Noise did not have a remarkable association with joint awareness or clinical outcomes as assessed by FJS-12, KSS-2011, or ROM. Some knees exhibited greater postoperative ROM and KSS-2011 with more frequent noise perception.

## Data Availability

The datasets used and/or analysed during the current study are available from the corresponding author on reasonable request.

## Abbreviations

ANOVA: Analysis of Variance
BCS: Bi-cruciate Stabilized
BMI: Body Mass Index
CR: Cruciate Retaining
CS: Cruciate Sacrificed
FJS-12: Forgotten Joint Score—12
KOOS: Knee Injury and Osteoarthritis Outcome Score
KSS-2011: 2011 Knee Society Knee Scoring System
OKS: Oxford Knee Score
PROM: Patient-Reported Outcome Measure
PS: Posterior Stabilized
ROM: Range of Motion
S.D.: Standard Deviation
S.E.: Standard Error
TKA: Total Knee Arthroplasty
WOMAC: the Western Ontario and McMaster Universities Osteoarthritis Index
